# Fatal multiple organ dysfunction caused by commensal bacteria of urogenital tract infection in adult lung transplant recipients: two case reports

**DOI:** 10.1186/s12985-022-01958-0

**Published:** 2023-02-08

**Authors:** Manman Tian, Dongsheng Han, Subo Ma, Tingting Liu, Wu Yang, Xia Zheng

**Affiliations:** 1 Department of Critical Care Units, The First Affiliated Hospital, Zhejiang University School of Medicine, 79 Qingchun Road, Hangzhou, 310003 China; 2 Department of Laboratory Medicine, The First Affiliated Hospital, Zhejiang University School of Medicine, Key Laboratory of Clinical In Vitro Diagnostic Techniques of Zhejiang Province and Institute of Laboratory Medicine, Zhejiang University, 79 Qingchun Road, Hangzhou, 310003 China

**Keywords:** Ureaplasma urealyticum, Herpes simplex virus type-2, Commensal bacteria of the urogenital tract, Lung transplantation, Next-generation sequencing

## Abstract

**Background:**

Infection following lung transplantation has been the focus of clinical concerns. The colonization rate of commensal bacteria of the urogenital tract, including *Mycoplasma hominis*, *Ureaplasma* urealyticum (UU), and *herpes simplex virus type-2 (HSV-2*), is high, which may cause secondary infection after transplantation.

**Case presentation:**

Twenty-three-year-old and 67-year-old women underwent lung transplantation for different causes. Shortly after the operation, they developed perineal skin ulcers, hypoxia, and intractable epilepsy. Subsequent computed tomography (CT) of the chest showed lung consolidation, and cranial CT showed shallowing sulci and gyri. *UU* and HSV-2 were detected in bronchoalveolar lavage fluid by next-generation sequencing, and HSV-2 was shown in the cerebrospinal fluid of both patients. Despite active treatment, both suffered irreversible brain function damage within 72 h of the seizure.

**Conclusions:**

Clinicians should know that commensal bacteria of urogenital tract infections can lead to fatal multiple organ dysfunction after lung transplantation.

**Supplementary Information:**

The online version contains supplementary material available at 10.1186/s12985-022-01958-0.

## Background

Lung transplantation is the ultimate and often last treatment option for those with various end-stage lung diseases. The number of lung transplants worldwide continues to grow. Adults who underwent primary lung transplantation had a median survival of 6.7 years [1]. The double lung transplantation survival rate was 84% at 1 year, 63% at 3 years, 61% at 5 years, and 48% at 10 years [2]; compared with other solid organ transplants, the mortality rate continues to be high [3]. Physicians need to attach great importance to the cognitive management of perioperative infections in lung transplants that involve multiple sites, and adverse outcomes due to commensal bacteria in the urogenital tract have received increasing attention in recent years. Once it occurs, the risk of death is substantial. Bacteria such as *Mycoplasma hominis*, *Ureaplasma urealyticum* (*UU*), and *herpes simplex virus type* 2 (HSV-2) are commensal bacteria in the urogenital tract that can coexist with the body and become pathogenic in males and females [4, 5]. Many donors and recipients of lung transplants may carry these germline coinfections and become secondarily infected after transplantation. Immunocompromised patients are susceptible to infection, especially those with solid organ transplantation, acquired immune deficiency syndrome, and hematological malignancies [6]. Several recent studies have found the rapid progression of hyperammonemia and high mortality after lung transplantation. *UU* infection could cause hyperammonemia in patients after lung transplantation [3, 7, 8]. This study reports two cases of fatal multiple organ dysfunction following lung transplantation caused by genital commensal *UU* in combination with *HSV-2* infection.

## Methods

### Sample collection

Blood samples are obtained by a routine aseptic procedure. For blood, only plasma is collected for further testing. 1–2 mL CSF samples were obtained during the lumbar puncture examination. Bronchoalveolar lavage fluid was collected, and the end of the bronchoscope was wedged into the bronchial opening at the site of pulmonary infiltration under an aseptic operation. Sterile saline was injected into the bronchoscope biopsy at 37 °C, and 5 mL of BALF was aspirated and recovered. Low-speed centrifugation (1,500 g for 20 min) was performed for all specimens to remove human cells in the samples, including BAL, blood, and CSF. Samples were then homogenized using bead beating followed by DNA extraction using the QIAamp^®^ UCP Pathogen DNA Kit (Qiagen) following the manufacturer's instructions. Total RNA was extracted with a QIAamp® Viral RNA Kit (Qiagen), and ribosomal RNA was removed by a Ribo-Zero rRNA Removal Kit (Illumina).

### Library construction and sequencing

Libraries were constructed for the DNA and cDNA samples using a Nextera XT DNA Library Prep Kit (Illumina, San Diego, CA). The library quality was assessed by a Qubit dsDNA HS Assay kit followed by a High Sensitivity DNA kit (Agilent) on an Agilent 2100 Bioanalyzer. Library pools were loaded onto an Illumina Nextseq CN500 sequencer for 75 cycles of single-end sequencing to generate approximately 20 million reads for each library.

### Bioinformatics analyses

Trimmomatic was used to remove low-quality reads, adapter contamination, and duplicate reads. Clean reads were obtained after short reads (length ≤ 500 bp) and low-quality reads (mean q-score ≤ 8) removal by Kcomplexity. Human sequence data were identified and excluded by mapping to a human reference genome (hg 38) using Burrows-Wheeler Aligner software. The database consisted of about 13,000 genomes and microbial reads aligned with SNAP v1.0beta [9]. Virus-positive detection results (DNA or RNA viruses) were defined as the coverage of three or more non-overlapping regions on the genome. A positive detection was reported for a given species or genus if the reads per million (RPM) ratio or RPM-r was ≥ 5, where the RPM-r was defined as the RPM sample/RPMNC (i.e., the RPM corresponding to a given species or genus in the clinical sample divided by the RPM in the NC/negative control).

## Case report

### Case 1

**Fig. 1:**
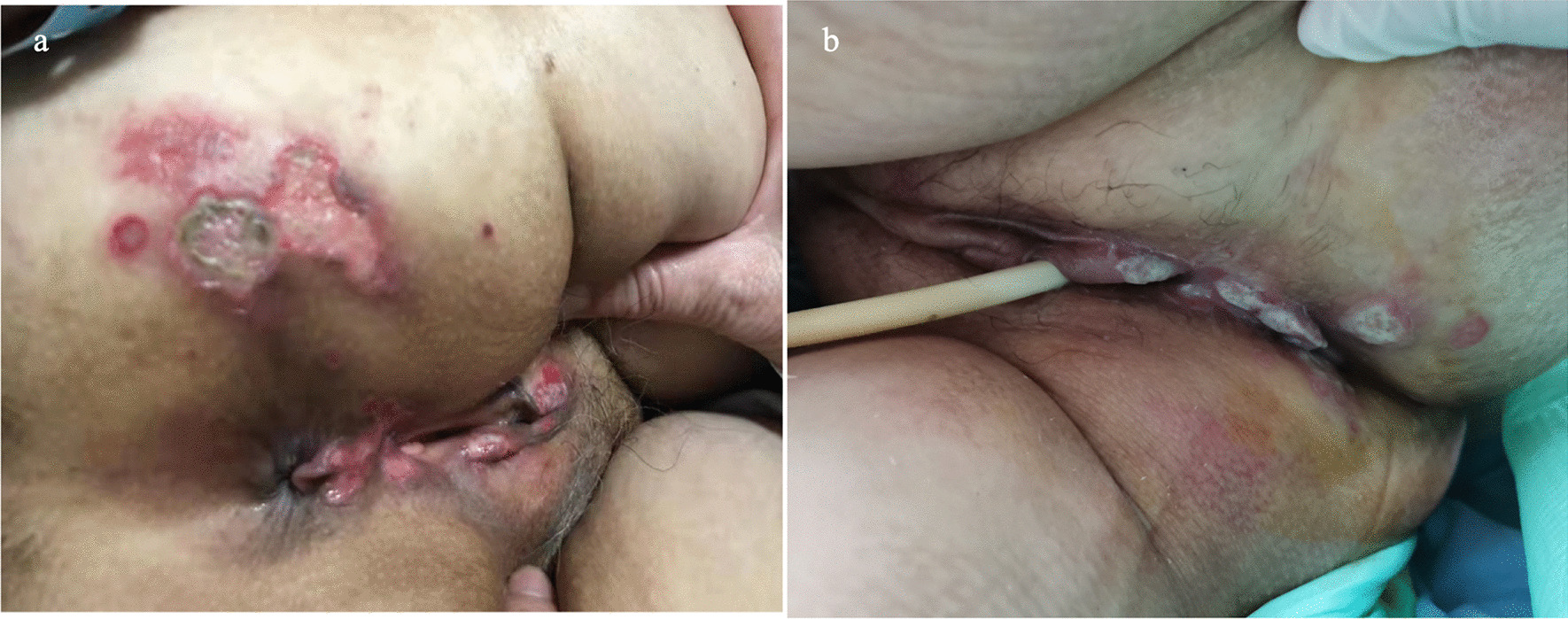
Perineum skin ulceration: **a** is CASE 1, **b** is CASE 2

The first case is a 23-year-old female who was poisoned by "glufosinate-ammonium" (containing paraquat), which resulted in pulmonary fibrosis, and she underwent double lung transplantation. After receiving the lung transplant, she was treated with the anti-rejection drugs tacrolimus and methylprednisolone. Because *Klebsiella* pneumoniae was cultured before the surgery, meropenem was given for anti-infection and voriconazole for anti-fungal infection. With a weak diaphragm, a tracheotomy had to be performed. On the post-operative day (POD) 26, round-shaped ulcers with distinct boundaries, known as perianal ulcers, emerged. 6 (Fig. 1a). After consultation with a dermatology specialist, she was given famciclovir to treat a suspected herpes virus infection.

**Fig. 2:**
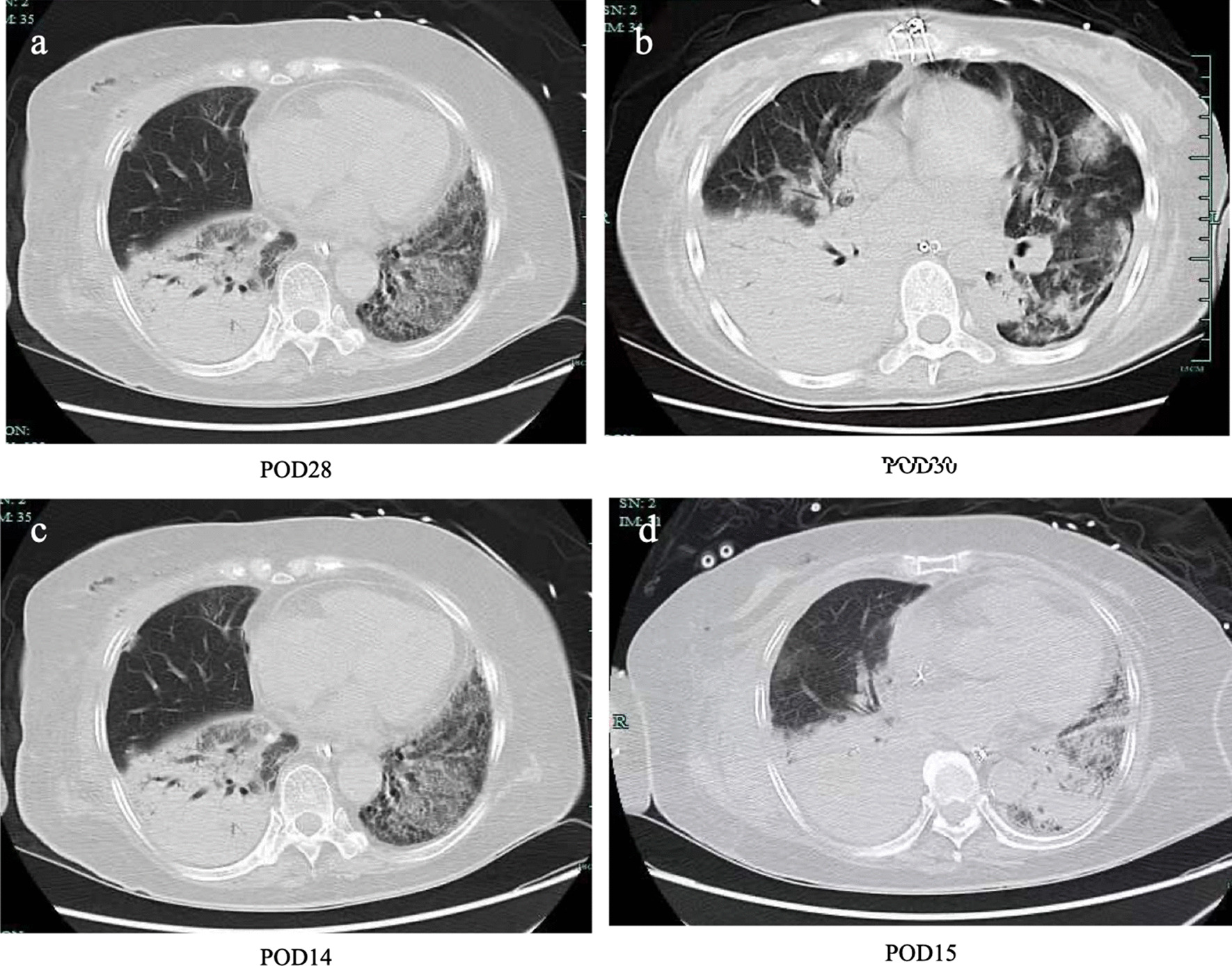
Pulmonary imaging showed pulmonary consolidation. **a** and **b** are the day after operation POD28 and POD30 of CASE1; **c** and **d** are the days after operation POD16 and POD17 of CASE2

**Fig. 3:**
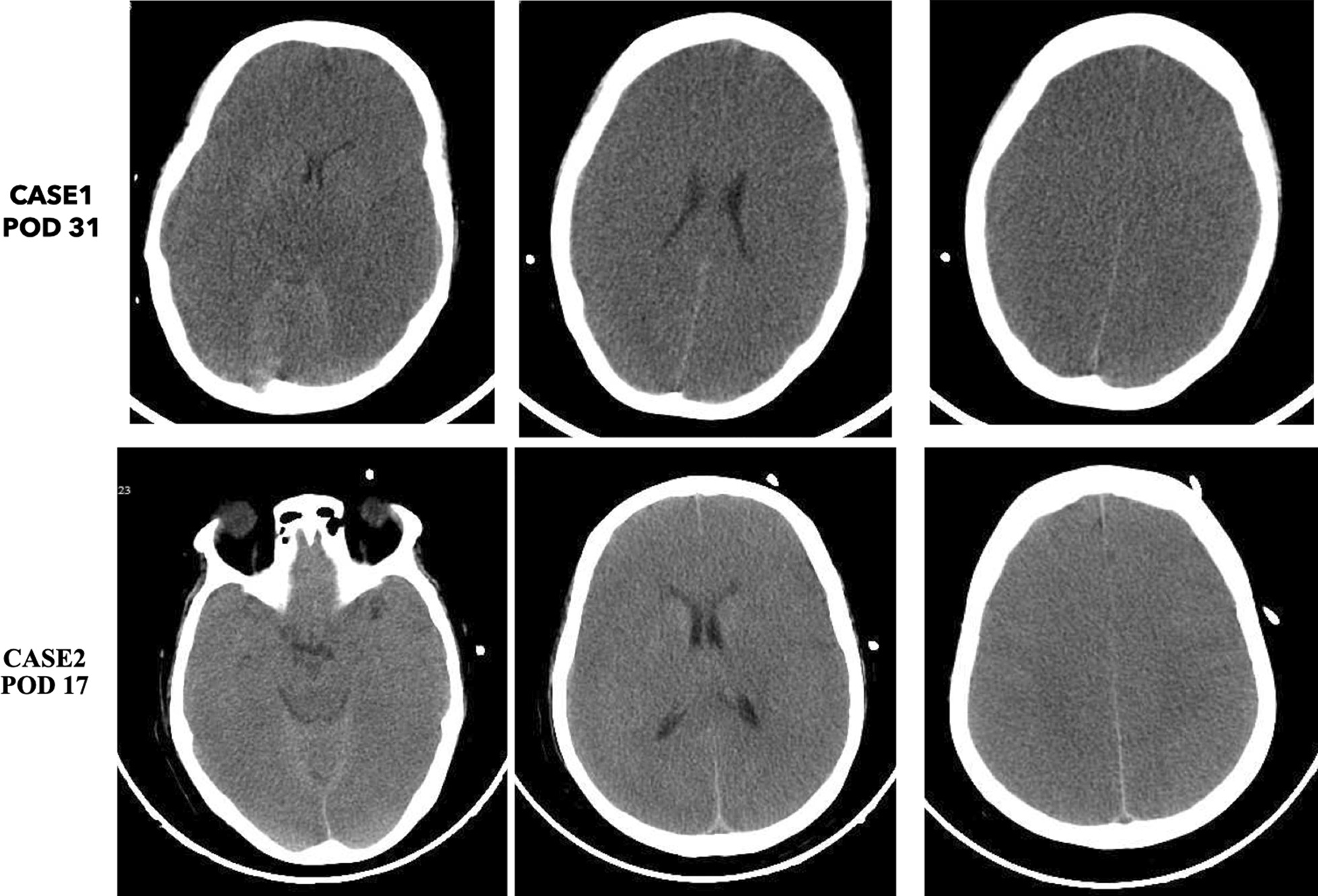
The cranial CT of CASE1 on POD31 (upper panel) and CASE2 on POD17 (lower panel). Cranial CT showed that the density of the brain parenchyma decreased slightly, and some sulci and gyri became shallow and disappeared

**Fig. 4:**
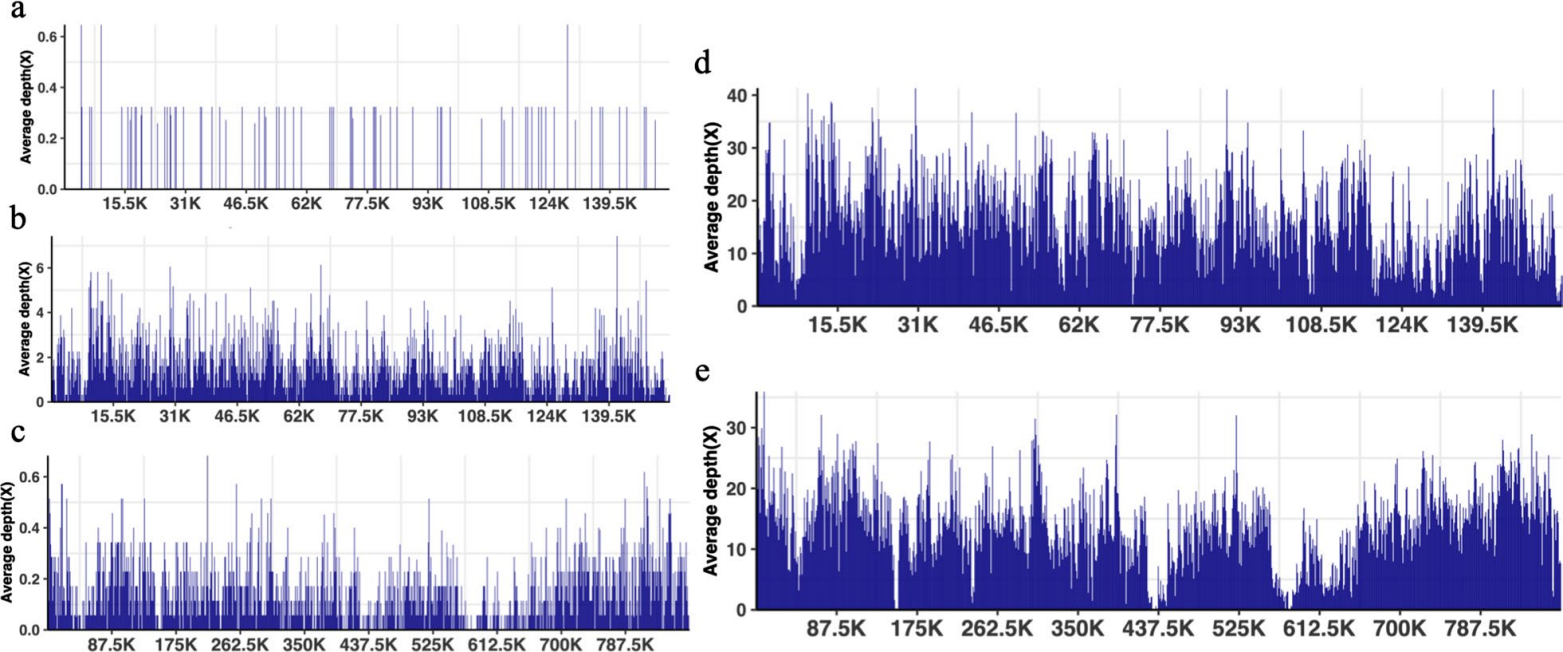
Results of next-generation sequencing in cerebrospinal fluid (CSF) and bronchoalveolar lavage fluid (BALF) in CASE 1 and CASE 2. **a** mNGS result of nucleotide sequences distributed along the genome of *HSV-2* in CSF of CASE 1 and the coverage of *HSV-2*: 2.48%. **b** mNGS result of nucleotide sequences distributed along the genome of *HSV-2* in BALF of CASE 1 and the coverage of *HSV-2*: 73.64%. **c** mNGS results of nucleotide sequences distributed along the genome of *Ureaplasma urealyticum* (*UU*) in BALF of CASE 1 and the coverage of *UU*: 14.53%. **d** mNGS result of nucleotide sequences distributed along the genome of *HSV-2* in BALF of CASE 2 and the coverage of *HSV-2*: 98.74%. **e** mNGS result of nucleotide sequences distributed along the genome of *UU* in BALF of CASE 2 and the coverage of *UU*: 94.86%

The patient's oxygenation index decreased gradually, and lung CT (Fig. 2a) showed consolidation, indicating aggravation of the patient's pulmonary infection. Therefore, she was treated in a prone position with cefoperazone/sulbactam, voriconazole, and vancomycin for anti-infection, but the patient's oxygenation still did not improve. Intractable epilepsy appeared on POD30. Midazolam was given intravenously, and propofol and remifentanil were continuously pumped, but it was still difficult to control, and bedside electroencephalogram (EEG) suggested seizures. Cranial CT showed that the density of the brain parenchyma decreased slightly, and some sulci and gyri became shallow and disappeared, suggesting brain edema and increased intracranial pressure (Fig. 3). One day later (POD31), the patient's condition further progressed. At approximately 5 a.m., the two pupils were deemed unequal in size. Emergency cranial CT suggested diffuse brain swelling. Next-generation sequencing (NGS) of cerebrospinal fluid (CSF) on POD31 revealed *HSV-2* infection (Fig. 4a). NGS results of bronchoalveolar lavage fluid (BALF) showed *Pseudomonas aeruginosa*, *HSV-2*, *UU* (Fig. 4b and c), and *human herpesvirus 5 (HSV-5)* infections. Lung CT results showed changes after lung transplantation, multiple infections in both lungs, and massive consolidation in the right lung (Fig. 2b). According to NGS results, vancomycin was stopped as antibiotics, levofloxacin and acyclovir were added to resist mycoplasma and virus infection, ceftazidime avibactam and voriconazole and amikacin atomization were continued to resist infection, and mannitol dehydration, levetiracetam, and sodium valproate were given to resist epilepsy. On POD31, her intractable epileptic seizures could not be controlled by the combination of various antiepileptic drugs, and the family member gave up on returning her home after being informed of her condition.

### Case 2

The second case is a 67-year-old female who underwent right lung transplantation due to pulmonary interstitial fibrosis. After the patient was admitted to critical care units, the physical examination showed skin ulcers of the perineum (Fig. 1b). The patient's family members described that the perineum skin ulcer existed before surgery. The patient was treated immediately with methylprednisolone, tacrolimus anti-rejection, cefoperazone/sulbactam, and voriconazole to prevent infection, and other supportive treatments were given. Her oxygenation worsened. On POD14, the prone position was changed to improve oxygenation, and CT showed lung consolidation (Fig. 2c and d). On POD16, she suddenly suffered intractable epileptic seizures. Midazolam, propofol, and remifentanil were intravenously administered, and she was immediately checked for no possible epilepsy caused by drugs. Cranial CT showed diffuse swelling of brain tissue and shallowing of the sulcus and cistern (Fig. 3). Mannitol infusion was instigated to reduce intracranial pressure. Analysis of the CSF revealed the following: karyocytes, 0/μl; red cells, 5/μl; protein, 38 g/l; glucose, 5.2 mmol/l; and chloride, 117 mmol/l. The intracranial pressure was 290 mmH2O. To rule out whether there was hypoperfusion in the brain, computerized tomography angiography (CTA) was performed to evaluate cerebral perfusion, and no obvious abnormality was found. Blood ammonia was monitored, and the blood ammonia level was 432 μmol/l. She received ornithine aspartate to reduce blood ammonia.

On POD18, NGS results of BALF fluid were reported: *UU*, HSV-2 (Fig. 4d and e), *Ureaplasma parvum*; NGS report of CSF was reported HSV-2. HSV-2 was treated with acyclovir antiviral and azithromycin for anti-UU infection. The urine, vaginal secretions, and skin secretions from the perineum ulcer were tested by pharyngeal swabs, and the follow-up results were all positive for UU antibodies. Monitoring the dynamic change in blood ammonia up to 927 μmol/l, intensive blood ammonia reduction treatment, rifaximin combined with ornithine aspartate, continuous bedside continuous renal replacement therapy (CRRT) blood ammonia reduction. However, the patient's condition was still worsening. On the evening of POD18, the transcranial Doppler (TCD) showed oscillating flow, which suggested that the patient's intracranial pressure was increased, cerebral blood flow was extremely poor, and the electroencephalogram (EEG) was a resting wave. A dynamic increase in neuron-specific enolase (NSE) was detected, which reflects the continuing aggravation of brain injury in patients. The patient's condition progressed rapidly from seizure to intracranial deterioration for approximately 48 h. On POD21, the patient gave up going home because of his critical condition and poor prognosis.

## Discussion

Comparing the two patients has the following common points: (1) both lung transplant patients need anti-rejection treatment. (2) Clinically, both patients had perineum skin ulcers, ulcers, decreased oxygenation, epilepsy, and lung consolidation on imaging. (3) NGS of BALF showed *UU*, *HSV-2* and *HSV-5*; NGS of cerebrospinal fluid showed *HSV-2*. (4) From the clinical course and prognosis, the disease progresses rapidly, and the mortality rate is high. Brain function damage is serious after the disease progress and epilepsy. Irreversible brain function damage occurs approximately 72 h after epilepsy. We attach great importance to commensal bacteria in the reproductive tract of lung transplant patients.

*HSV-2*, also known as genital herpes, is one of the most common sexually transmitted infections in the world. In 2016, an estimated 491.5 million people were living with HSV-2 infection [5]. *HSV-2* mainly causes herpes in reproductive organs, most of which are transmitted by sexual contact. The lesions were near the buttocks, penis, vagina, and cervix.

Primary infection may cause discomfort, fever, or localized glandular disease [10]. Two cases of infection after lung transplantation reported in this study had skin infection symptoms before the onset of the disease. One older woman had genital herpes before transplantation, and one young woman had genital herpes after transplantation.

*Mycoplasma hominis* and *UU* are also normal genital flora of many men and women who have sex. The proportion of *Mycoplasma hominis* colonization increases with the increase in sexual behavior, and the increase is faster in women than in men, indicating that women are more prone to colonization [11]. It has also been found that Ureaplasma is usually a symbiotic genitourinary pathogen [4], with up to 29% of males and 38–75% of females, leading to urinary tract infection, urethritis, cervicitis, pelvic inflammation [4], pneumonia [12], soft tissue infection [13], endocarditis [14], brain abscess [6] and other genital infections. In particular, it is more common in patients with low immune function. It was reported that the two cases were also female and accompanied by pulmonary infection.

Hyperammonemia syndrome is a rare but potentially fatal complication after lung transplantation. Mainly seen in lung transplant recipients [15]. Hyperammonemia was identified as a new psychiatric symptom after transplantation with blood ammonia > 200 μmol/l [16]. The cause is unclear, but there is evidence that it is related to *Ureaplasma* and *Mycoplasma hominis* infection. Therefore, the blood ammonia level should be monitored in cases of sudden changes in the consciousness of patients with depressed immune function. The study showed that the positive rate of *Mycoplasma* and *Ureaplasma* after lung transplantation was high (28.5%), and the study believed that the infection came from the donor. In addition, the study showed that the positive rate of *UU* was independently related to young female donors [7]. Hyperammonemia may occur in 1–4% of lung transplant recipients, with a mortality rate of 67% for 30 days after transplantation and 17% in patients with normal ammonia levels [17].

Patients with lung transplantation need to take anti-rejection drugs for a long time after surgery. *UU* appears in the alveolar lavage fluid, and local infection of genital pathogens leads to lung infection, which may be related to the damage of the lung directly exposed to systemic venous circulation and donor pulmonary lymphatic drainage after transplantation, which will increase the risk of pneumonia in the first 4 to 6 weeks after transplantation. The majority of patients with hyperammonaemia underwent bilateral lung transplantation. Whether this is a major risk factor compared with single lung transplantation and whether it is related to the increase in the pathogen load of bilateral donor lung block or the lack of pulmonary lymphatic vessels in both lungs remains to be determined [11].

Some studies reported *HSV-2* as the most frequent herpes virus-causing CNS infection [18]. *HSV-2* can cause adults with normal immune function to suffer from acute and subacute myelitis [19], brainstem encephalitis, encephalitis, and meningitis [20].

The mortality of lung transplantation combined with hyperammonemia is higher. If combined on this basis, the neurological symptoms of viral encephalitis are more obvious, the course of the disease is rapid, and the mortality is higher. In our two cases, Case 1 monitored the blood ammonia level, while Case 2 did not monitor the blood ammonia level because of an insufficient understanding of hyperammonemia after lung transplantation at that time.

The CT scanning features of patients with Mycoplasma pneumoniae pneumonia reported in the literature are nodular and tree bud-shaped shadows (patchy distribution) in the center of the lobule, lobular or segmental ground glass-like shadows or consolidation, and thickening of the bronchial vascular bundle, which can be combined with pleural effusion [21]. Other studies have shown that *UU* can cause respiratory diseases in neonates [12].

The lung images of the two patients with genital pathogen infection (*UU*, *HSV-2*) reported by this study have the same imaging characteristics of pulmonary consolidation. However, in addition to the above genital pathogen infection, the BALF of CASE1 patients had a high colony count of *Pseudomonas aeruginosa*. Because the number of cases was small, consolidation caused by the combination of bacteria could not be ruled out.

## Conclusion

After lung transplantation, commensal bacteria of urogenital tract infection can lead to fatal multiple organ dysfunction, such as skin ulcers, lung consolidation, and nervous system changes. Perhaps early screening of commensal bacteria in the urogenital tract for transplant donors and recipients can help doctors make an early diagnosis and treatment, which may improve the survival rate of such patients.

## Supplementary Information

**Additional file 1. Additional Fig. 1.** Summary of main treatment, major events, and metagenomics results during the whole hospitalization course of CASE2. **Additional Fig. 2.** The Cranial CT of CASE1. **Additional Fig. 3.** Cranial CT of CASE2. **Additional Fig. 4.** Results of next-generation sequencing (NGS) in CASE 1. **Additional Fig. 5.** Results of next-generation sequencing (NGS) in CASE 2. **Additional Table. 1.** Comparison between CASE1 and CASE2 of commensal bacteria of urogenital tract infection.

## Data Availability

The original contributions presented in the study are included in the article/Additional file 1, and further inquiries can be directed to the corresponding authors.

## Abbreviations

*UU*: *Ureaplasma urealyticum*
*HSV-2*: *Herpes simplex virus type-2*
*HSV-5*: *Herpes simplex virus type 5*
NGS: Next-generation sequencing
CSF: Cerebrospinal fluid
BALF: Bronchoalveolar lavage fluid
CTA: Computed tomography angiography
CT: Computed tomography
EEG: Electroencephalogram
CRRT: Continuous bedside continuous renal replacement therapy
TCD: Transcranial Doppler
