# Supplementary material for: Fatal multiple organ dysfunction caused by commensal bacteria of urogenital tract infection in adult lung transplant recipients: two case reports
Source: Virol J. 2023 Feb 8;20:24. doi: 10.1186/s12985-022-01958-0 (PMC9906855; doi:10.1186/s12985-022-01958-0)
Supplement: Supplementary file 1 — Additional file 1. Additional Fig. 1. Summary of main treatment, major events, and metagenomics results during the whole hospitalization course of CASE2. Additional Fig. 2. The Cranial CT of CASE1. Additional Fig. 3. Cranial CT of CASE2. Additional Fig. 4. Results of next-generation sequencing (NGS) in CASE 1. Additional Fig. 5. Results of next-generation sequencing (NGS) in CASE 2. Additional Table. 1. Comparison between CASE1 and CASE2 of commensal bacteria of urogenital tract infection. [file 12985_2022_1958_MOESM1_ESM.docx]

**Fatal multiple organ dysfunction caused by commensal bacteria of urogenital tract infection in adult lung transplant recipients: two case reports(Additional file)**

**Additional Figure and table about CASE1 and CASE2:**

**Additional Fig. 1**

**Summary of main treatment, major events, and metagenomics results during the whole hospitalization course of CASE2.** Abbreviations: BAL, bronchoalveolar lavage; CSF, cerebrospinal fluid.


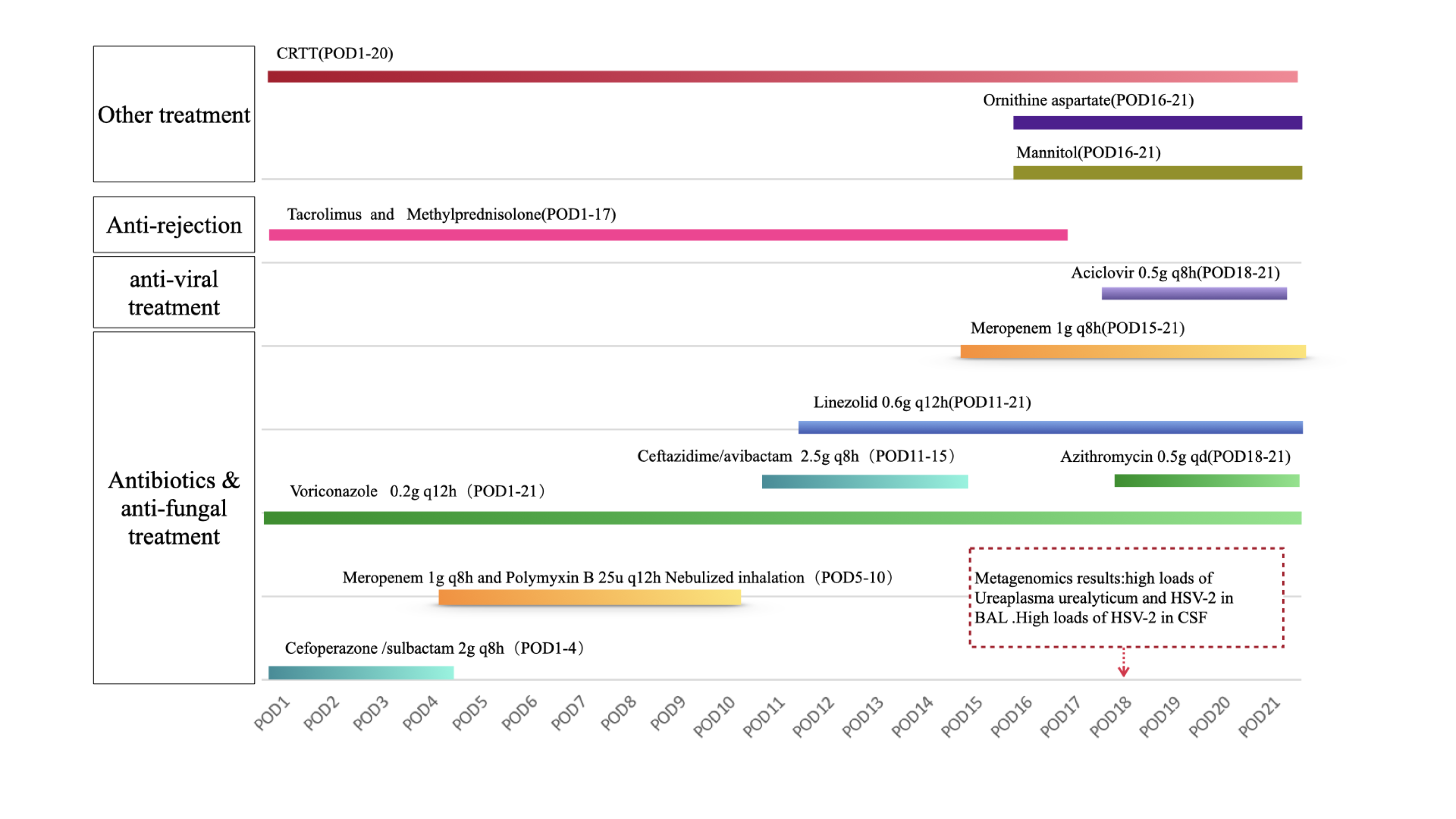


**Additional Fig. 2**

**The Cranial CT of CASE1**. The days after operation POD30 and POD31 showed that the density of brain parenchyma decreased slightly, and some sulci and gyri became shallow and disappeared.


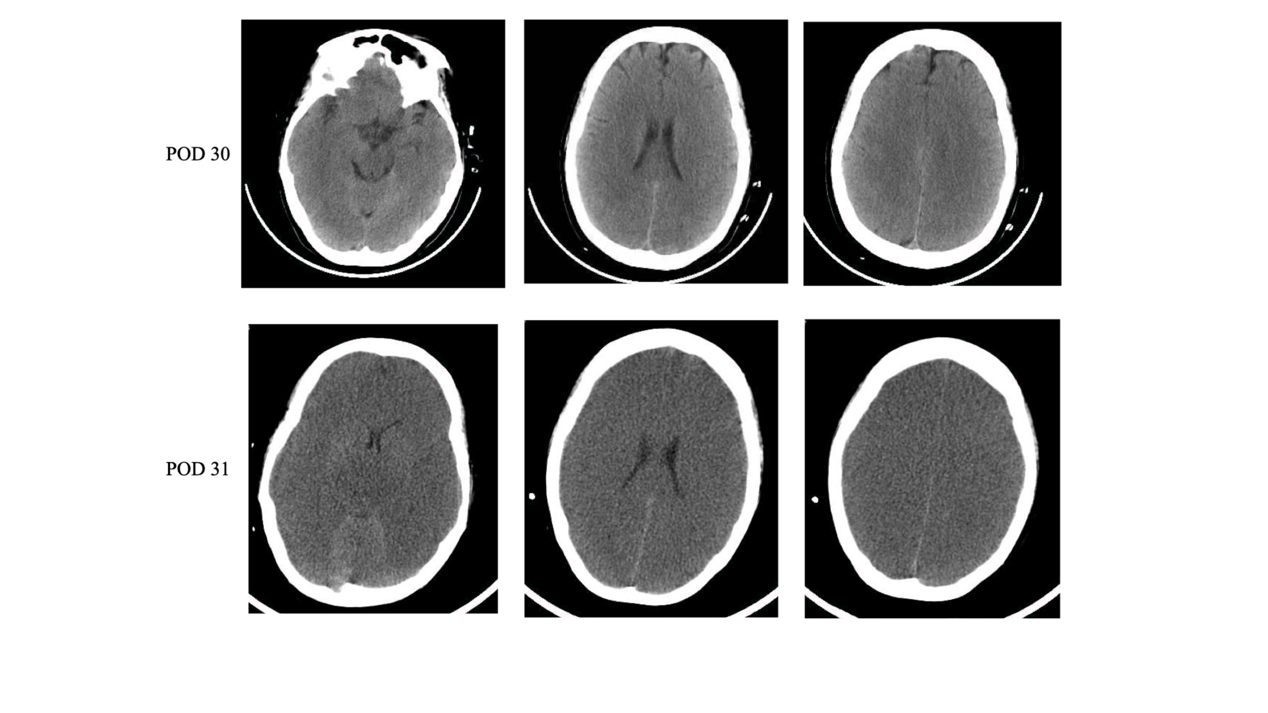


**Additional Fig. 3**

**Cranial CT of CASE2.** There showed no brain tissue swelling on POD5. There showed that the density of brain parenchyma decreased slightly, and some sulci and gyri became shallow and disappeared on POD16 and POD17 .


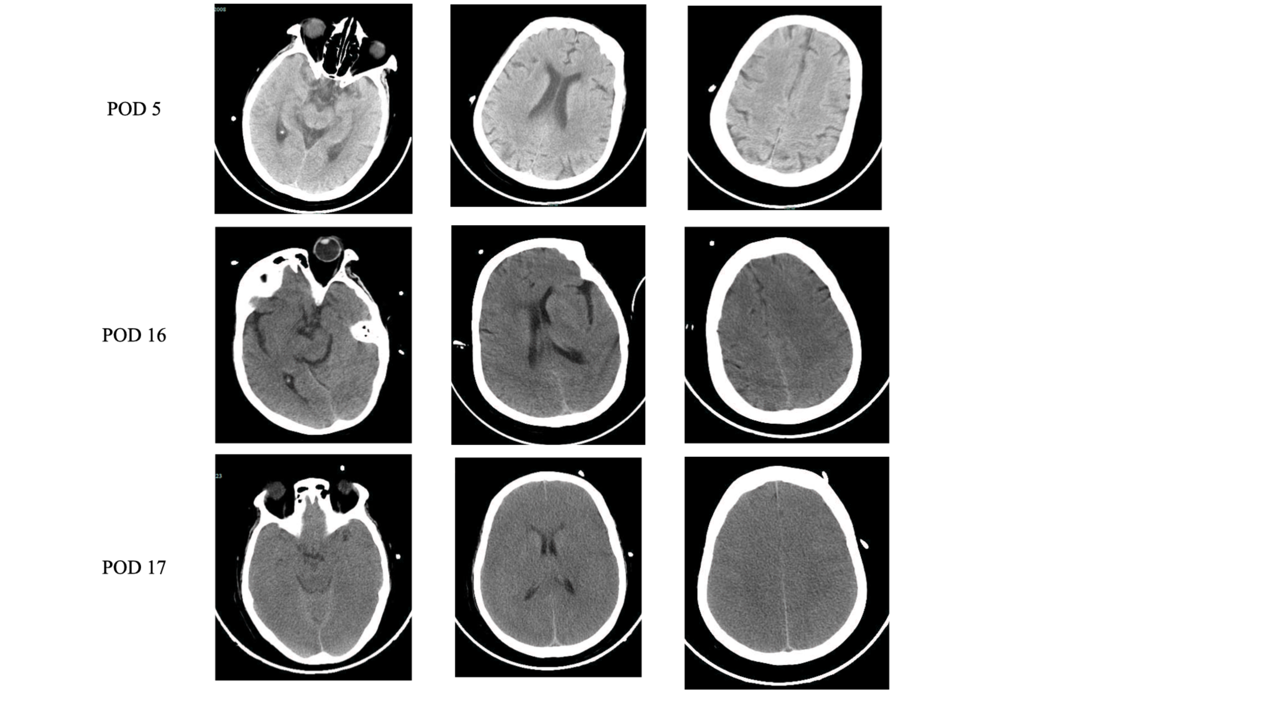


**Additional Fig. 4**

**Results of next-generation sequencing(NGS) in CASE 1**. a. Results of NGS in cerebrospinal fluid (CSF). b. Results of NGS in bronchoalveolar lavage fluid (BALF)


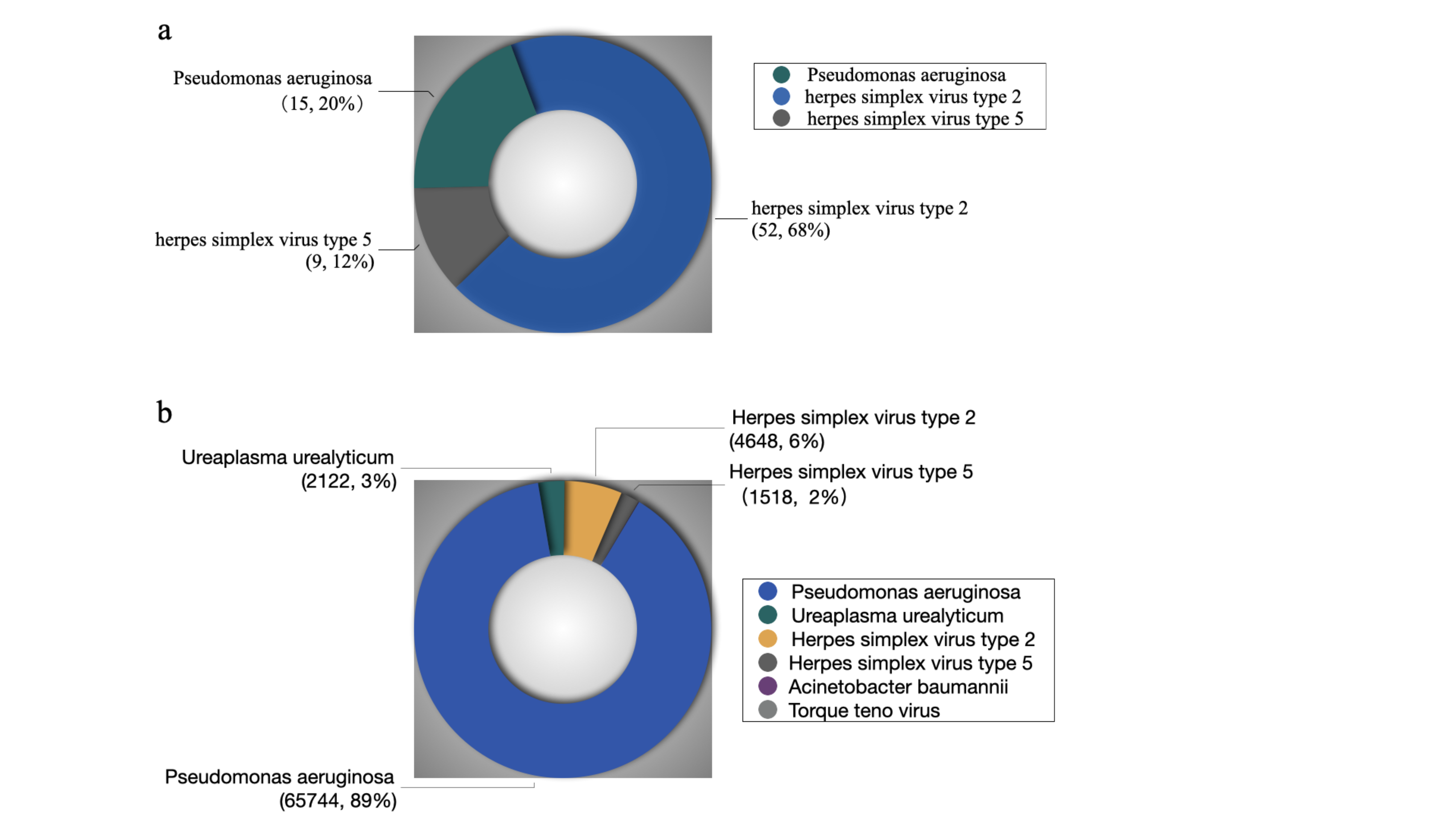


**Additional Fig. 5**

**Results of next-generation sequencing(NGS) in CASE 2.**  a. Results of NGS in cerebrospinal fluid (CSF). b. Results of NGS in bronchoalveolar lavage fluid (BALF)


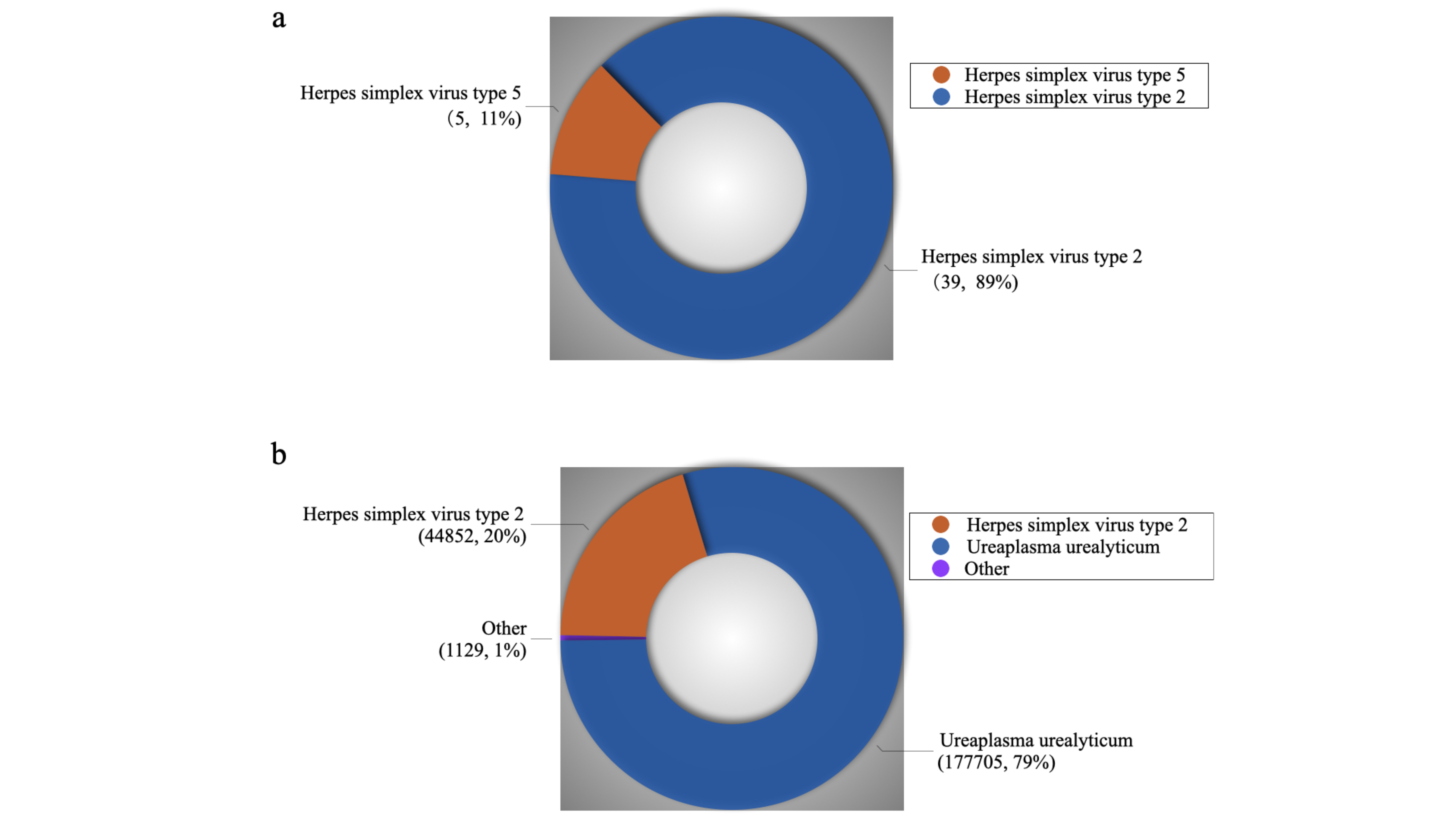


**Additional Table. 1**

**Comparison between CASE1 and CASE2 of commensal bacteria of urogenital tract infection.** Neuron specific enolase: NSE; Electroencephalogram: EEG；Computerized tomography: CT; Computerized tomography angiography: CTA; Transcranial doppler: TCD.

|  | Skin ulceration | Pulmonary CT | Nervous system | | | | | | |
| --- | --- | --- | --- | --- | --- | --- | --- | --- | --- |
|  |  |  | Epilepsy | Blood ammonia | NSE | Cranial CT | Cranial CTA | EEG | TCD |
| Case1 | + | consolidation | + | / | / | Edema | Normal | Epileptiform waves | / |
| Case2 | + | consolidation | + | ↑ | ↑ | Edema | / | resting wave | oscillating flow |
